# The morphology of the lumbar vertebrae: a systematic review with meta-analysis of 1481 individuals with implications for spine surgery

**DOI:** 10.1007/s00276-024-03509-4

**Published:** 2024-12-06

**Authors:** Michał Bonczar, Jan Koszewski, Wiktor Czarnota, Martyna Dziedzic, Patryk Ostrowski, Kamil Możdżeń, Agnieszka Murawska, Paweł Hajdyła, Andrzej Walocha, Ewa Walocha, Jerzy Walocha, Mateusz Koziej

**Affiliations:** 1 Department of Anatomy, Jagiellonian University Medical College Cracow, Mikołaja Kopernika 12, 33-332 Kraków, Poland; 2 Youth Research Organization, Youthoria, Kraków, Poland

**Keywords:** Lumbar spine, Lumbar vertebrae, Anatomy, Surgery, Orthopaedics

## Abstract

**Introduction:**

The aim of the present meta-analysis was to provide the most up-to-date and evidence-based results regarding the morphometric properties of the lumbar vertebrae.

**Methods:**

Medical databases such as PubMed, Scopus, Embase, Web of Science, Google Scholar, and Cochrane Library were searched through.

**Results:**

The results of this meta-analysis were established based on a total of 1481 patients. New results were established in 27 categories for each lumbar vertebra separately. The findings from this study reveal that the width of the spinal canal progressively increases towards the lower end of the lumbar spine (L1 = 22.04 mm, L5 = 26.46 mm). Additionally, the transverse processes exhibit a similar trend, widening as they approach the lower lumbar vertebrae (L1 = 68.08 mm, L5 = 85.91 mm). The pedicle height decreased from L1 to L4, with an increase observed at L5 (14.73 mm). No significant differences were observed between the measurements of the left and right pedicles.

**Conclusion:**

The presented results provide physicians with normative morphometric data on the lumbar vertebrae. Having adequate knowledge of the anatomy of the lumbar vertebrae may be of immense use for surgeons performing various spinal surgeries, such as pedicle screw fixation, percutaneous endoscopic transforaminal discectomy, or lumbar disc replacement.

## Introduction

The lumbar spine, also known as the lower back, consists of the five lumbar vertebrae located in the lower region of the vertebral column. These vertebrae are the largest and strongest in the spinal column, as they bear a significant amount of weight and provide stability and support to the upper body. Each lumbar vertebra is numbered L1 to L5, starting from the top. They are characterized by several distinctive features, including size and shape, the structure of the vertebral body, and their spinous processes, amongst others [3, 20, 21]. The anterior aspect of the vertebral body is taller than the posterior aspect, giving it a wedge-shaped appearance. Moreover, the lumbar spinous processes, i.e., the bony projections that extend backward from the posterior aspect of each vertebra, are relatively short and typically project straight posteriorly. The articular processes of the lumbar vertebrae extend vertically, initially having sagittal-oriented articular facets. Compared to thoracic vertebrae, the foramen of the lumbar vertebrae is larger, although it is still smaller than the foramen of cervical vertebrae. The foramen itself has a triangular shape [7, 21].

In anatomy textbooks, the term “norm” is associated with such concepts as general patterns, rules, or canon [39]. Numerous papers about the morphology of the lumbar spine vertebrae have been conducted. The majority of studies have employed either cadaveric or osteological specimens or have utilized advanced imaging techniques like computed tomography (CT) or magnetic resonance imaging (MRI) [7, 18, 38]. Studies involving cadaveric specimens have employed measurement tools such as Vernier calipers, digital calipers, or a 3-dimensional digitizing apparatus [10, 33]. Acquiring sufficient knowledge about the normative anatomy of the spinal column is imperative for ensuring precise diagnosis and effective treatment of spinal pathologies, such as congenital and acquired types of spinal stenosis (). Therefore, the aim of the present meta-analysis was to provide the most up-to-date and evidence-based results regarding the morphometric properties of the lumbar vertebrae.

## Materials and methods

### Search strategy

In order to perform this meta-analysis, a systematic search was conducted in which all articles regarding the anatomy of the lumbar vertebrae were searched for. Medical databases such as PubMed, Scopus, Embase, Web of Science, Google Scholar, and Cochrane Library were searched through. The overall search process was conducted in 3 stages. (1) In the first step, all mentioned medical databases were searched using the following search terms: (lumbar AND (vertebrae OR vertebral) AND anatomy) OR (lumbar AND spine AND anatomy). Neither date, language, article type, nor text availability conditions were applied. (2) Subsequently, the mentioned databases were searched through once again using another set of search phrases: (a) ((lumber spine[Title/Abstract]) OR (lumbar vertebrae[Title/Abstract])) AND (anatomy[Title/Abstract]) ; (b) ((lumber spine[Title/Abstract]) OR (lumbar vertebrae[Title/Abstract])) AND (morphology[Title/Abstract]) ; (c) ((lumber spine[Title/Abstract]) OR (lumbar vertebrae[Title/Abstract])) AND (dimensions[Title/Abstract]) ; (d) ((lumber spine[Title/Abstract]) OR (lumbar vertebrae[Title/Abstract])) AND (size[Title/Abstract]) ; (e) ((lumber spine[Title/Abstract]) OR (lumbar vertebrae[Title/Abstract])) AND (width[Title/Abstract]) ; (f) ((lumber spine[Title/Abstract]) OR (lumbar vertebrae[Title/Abstract])) AND (length[Title/Abstract]) ; (g) ((lumber spine[Title/Abstract]) OR (lumbar vertebrae[Title/Abstract])) AND (variation[Title/Abstract]) ; (h) ((lumber spine[Title/Abstract]) OR (lumbar vertebrae[Title/Abstract])) AND (morphometry[Title/Abstract]). (3) Furthermore, a manual search was also conducted throughout all references from the initial submitted studies. The Preferred Reporting Items for Systematic Reviews and Meta-Analyses (PRISMA) guidelines were followed. Additionally, The Critical Appraisal Tool for Anatomical Meta-analysis (CATAM) and Anatomical Quality assessment Tool (AQUA) were used to provide the highest quality findings [9, 13].

### Eligibility assessment and data extraction

**Fig. 1:**
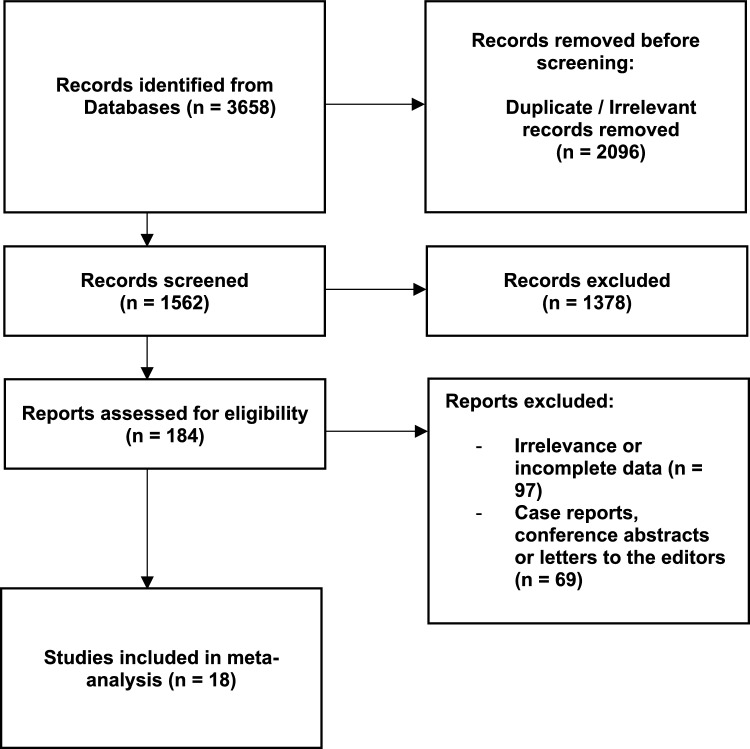
Flow diagram presenting process of collecting data included in this meta-analysis

The inclusion criteria were established as follows: original articles with extractable data on the overall anatomy of the lumbar vertebrae. The exclusion criteria involved conference reports, case reports, case series, reviews, letters to the editor, patients with a noticeable pathology that could potentially distort the studied anatomy, and studies with no relevant or incompatible data. The systematic search was performed by two independent researchers. A total of 3658 articles were initially evaluated by two independent reviewers. After removing duplicate or irrelevant records (*n* = 2096), a total of 1562 articles were screened. Finally, 18 articles matched the required criteria and were used in this meta-analysis [1, 6, 7, 10, 15, 16, 18, 20, 22, 23, 25, 27, 30, 32–34, 36, 38]. The overall process of data collection can be found in Fig. 1. Characteristics of submitted articles can be found in Table 1.

**Table 1:** Characteristics of the studies submitted to this meta-analysis

First author	Year of publication	Continent	Country	Method	Research group	Mean Age	Mean Weight (kg)	Mean Height (m)
Naidoo N.	2022	Asia	United Arab Emirates	Magnetic Resonance Imaging	50	–	–	–
Irshad F.	2022 (a)	Asia	Pakistan	Computed Tomography	26	Females: 45 ± 7.3 Males: 48 ± 9.7	–	–
Irshad F.	2022 (b)	Asia	Pakistan	Radiograph	81	Females: 29 ± 6.9 Males: 34 ± 7.6	–	–
Cook W.H.	2021	Oceania	New Zealand	Computed Tomography	196	42 ± 19.5	**–**	–
Singh V.	2021	Asia	India	Computed Tomography	302	–	–	–
Morita K.	2021	Asia	Japan	Computed Tomography	227	Females: 66.5 ± 15.2 Males: 67 ± 13.1	–	–
Agichani S.	2018	Asia	India	Radiograph	120	–	–	–
Gomez-Olivencia A.	2017	Europe	Spain	Digital Callipers	73	–	–	–
Wang Y.	2015	Asia	China	Computed Tomography	5	–	–	–
Mahato N.K.	2011	Asia	India	Radiograph	50	32 ± 7.82	–	–
Been E.	2010	Asia	Israel	Vernier Caliper	97	–	–	–
Tan S. H.	2003	Asia	Singapore	Three-dimensional Digitiser	10	65.7	61.6	1.66
Kadioglu H.	2003	Asia	Turkey	Computed Tomography	29	35	–	–
Tan S. H.	2001	Asia	Singapore	Three-dimensional Digitiser	12	67	62	1.66
Wolf A.	2001	Asia	Israel	Computed Tomography	55	57.64 ± 17.84	–	–
Robertson P.	2000	Oceania	New Zealand	Radiograph	10	–	–	–
Zhou S. H.	2000	Europe	UK	Computed Tomography	126	Females: 49 Males: 50	–	–
Panjabi M.	1992	Asia	India	Radiograph	12	46.3	67.8	1.678

Two independent reviewers extracted data from qualified studies. They collected qualitative data, such as the year of publication, country, and continent. Subsequently, they gathered quantitative data regarding the anatomical, especially morphometrical, features of the lumbar vertebrae. Any discrepancies between the studies identified by the two people were resolved by contacting the authors of the original studies wherever possible or by consensus with a third person.

### Statistical analysis

To perform this meta-analysis, STATISTICA version 13.1 software (StatSoft Inc., Tulsa, OK, USA), MetaXL version 5.3 software (EpiGear International Pty Ltd, Wilston, Queensland, Australia), and Comprehensive Meta-analysis version 4.0 software (Biostat Inc., Englewood, NJ, USA) were applied. A random effects model was used. The Chi-square test and the I-squared statistic were chosen to assess the heterogeneity among the studies [12, 14]. P-values and confidence intervals were used to determine the statistical significance between the studies. A p-value lower than 0.05 was considered statistically significant. In the event of overlapping confidence intervals, the differences were considered statistically insignificant. I-squared statistics were interpreted as follows: values of 0–40% were considered as “might not be important”, values of 30–60% were considered as “might indicate moderate heterogeneity”, values of 50–90% were considered as “may indicate substantial heterogeneity”, and values of 75–100% were considered as “may indicate substantial heterogeneity.” The overall analysis could have been performed as the results obtained using different methods did not differ statistically significantly from one another (*p* > 0.05).

## Results

**Fig. 2:**
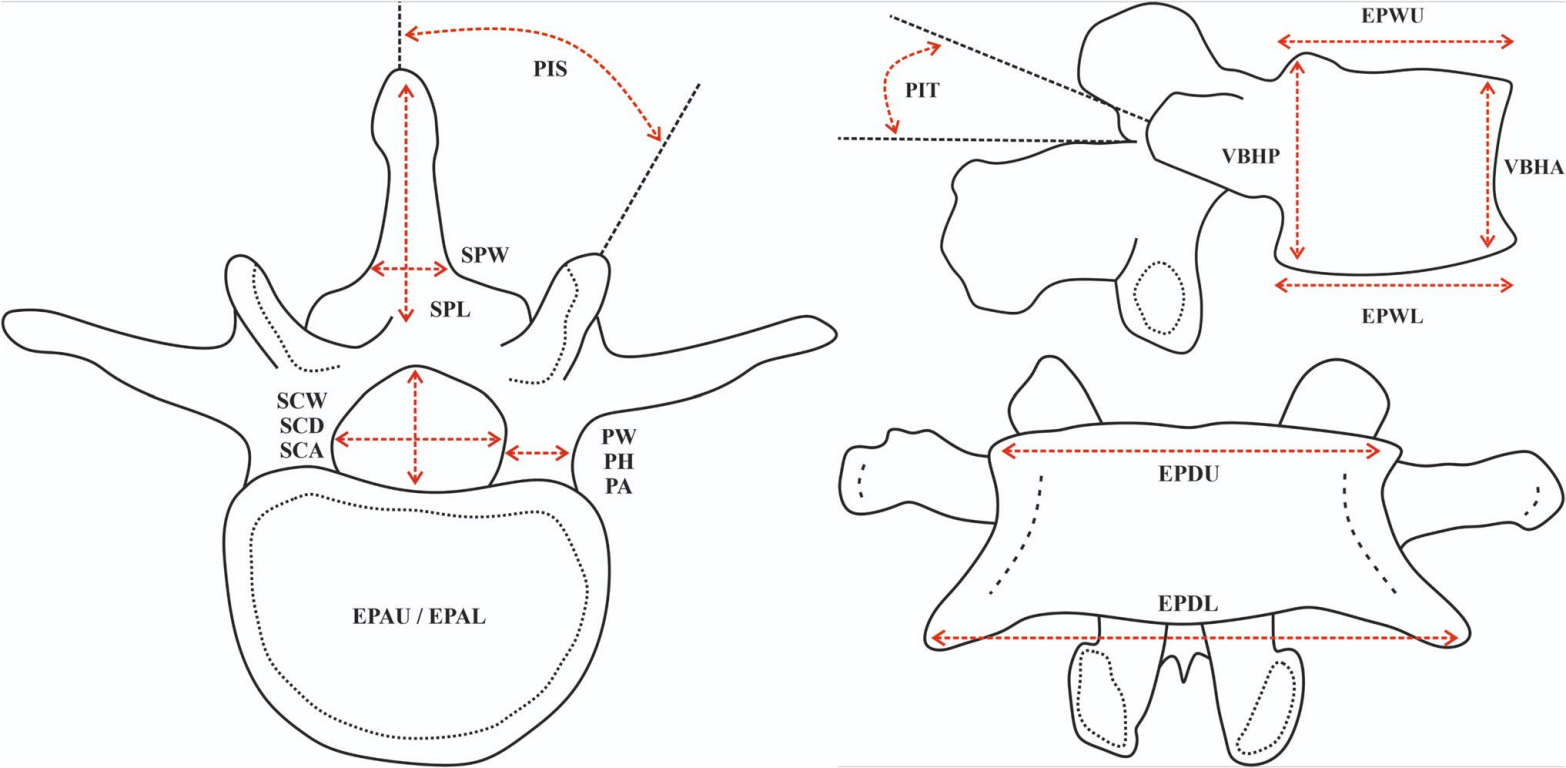
A scheme, graphically presenting some of the categories of the results of this meta-analysis. EPDU - End-plate depth upper. EPWU - End-plate width upper. EPDL - End-plate depth lower. EPWL - End-plate depth lower. VBHA - Vertebral body height anterior. VBHP - Vertebral body height posterior. EPAU - End-plate area upper. EPAL - End-plate area lower. SCW - Spinal canal width. SCD - Spinal canal depth. SCA - Spinal canal area. PW - Pedicle width. PH - Pedicle height. PA - Pedicle area. PIS - Pedicle inclination saggital plane. PIT - Pedicle inclination transcerse plane. SPL - Spinous process length. SPW - Spinous process width

The results of this meta-analysis were established based on 1481 patients. New results were established in 27 categories for each lumbar vertebra separately. Additionally, a sexual dimorphism analysis was performed in some of the categories in which it was possible. Most of the parameters in which the new results were calculated are presented in a scheme in Fig. 2.

L1 Dimensions: The mean depth of the upper part of the end-plate was found to be 31.04 mm (SE = 1.07), while its width was set at 41.09 mm (SE = 1.60). The mean depth of the lower part of the end-plate was found to be 32.27 mm (SE = 1.20), while its width was set at 43.97 mm (SE = 1.74). The heights of the anterior and posterior vertebral body parts were established to be 24.54 mm (SE = 0.96) and 26.57 mm (SE = 1.06), respectively. The mean spinal canal width was set to be 22.04 mm (SE = 0.91). The mean spinal canal area was set to be 251.01 mm^2^ (SE = 16.47). All the results mentioned above and more detailed ones regarding the L1 vertebra can be found in Table 2.

**Table 2:** Statistical results of this meta-analysis regarding the L1 dimensions

L1 dimensions	Mean	Standard Error	Variance	Lower Limit	Upper Limit	Z-Value	*p*-Value
End-plate depth upper (mm)	31.04	1.07	1.15	28.94	33.14	28.96	0.00
End-plate width upper (mm)	41.09	1.60	2.55	37.96	44.22	25.73	0.00
End-plate depth lower (mm)	32.27	1.20	1.45	29.91	34.63	26.82	0.00
End-plate width lower (mm)	43.97	1.74	3.02	40.56	47.38	25.29	0.00
Vertebral body height anterior (mm)	24.54	0.96	0.92	22.67	26.42	25.66	0.00
Vertebral body height posterior (mm)	26.57	1.06	1.12	24.50	28.65	25.11	0.00
End-plate area upper (mm^2^)	932.26	56.46	3187.29	821.61	1042.92	16.51	0.00
End-plate area lower (mm^2^)	1039.83	55.77	3109.75	930.53	1149.13	18.65	0.00
End-plate inclination transverse plane upper (degrees)	2.18	0.23	0.05	1.74	2.63	9.63	0.00
End-plate inclination transverse plane lower (degrees)	−0.55	1.69	2.87	−3.87	2.77	−0.33	0.75
Spinal canal width (mm)	22.04	0.91	0.83	20.25	23.82	24.17	0.00
Spinal canal depth (mm)	16.02	1.07	1.14	13.93	18.12	14.99	0.00
Spinal canal area (mm^2^)	251.01	16.47	271.30	218.73	283.29	15.24	0.00
Pedicle width (mm)	7.51	0.58	0.34	6.36	8.65	12.85	0.00
Pedicle height (mm)	15.40	0.28	0.08	14.84	15.95	54.37	0.00
Pedicle width right (mm)	7.30	0.50	0.25	6.32	8.28	14.61	0.00
Pedicle height right (mm)	15.71	0.74	0.55	14.26	17.17	21.13	0.00
Pedicle width left (mm)	7.73	0.51	0.26	6.73	8.73	15.17	0.00
Pedicle height left (mm)	15.47	0.68	0.46	14.13	16.80	22.76	0.00
Pedicle area right (mm^2^)	78.28	5.15	26.54	68.18	88.37	15.20	0.00
Pedicle area left (mm^2^)	78.29	5.27	27.82	67.95	88.63	14.84	0.00
Pedicle inclination sagittal plane right (degrees)	10.31	1.21	1.47	7.94	12.68	8.52	0.00
Pedicle inclination transverse plane right (degrees)	−0.44	0.93	0.87	−2.26	1.39	−0.47	0.64
Pedicle inclination sagittal plane left (degrees)	2.62	2.31	5.34	−1.91	7.15	1.13	0.26
Pedicle inclination transverse plane left (degrees)	−0.27	1.03	1.07	−2.29	1.76	−0.26	0.80
Spinous process length (mm)	59.94	4.57	20.86	50.99	68.89	13.12	0.00
Transverse process width (mm)	68.08	4.59	21.06	59.08	77.07	14.83	0.00

L2 Dimensions: The mean depth of the upper part of the end-plate was found to be 32.12 mm (SE = 1.09), while its width was set at 42.98 mm (SE = 1.57). The mean depth of the lower part of the end-plate was found to be 32.91 mm (SE = 1.17), while its width was set at 46.10 mm (SE = 2.12). The heights of the anterior and posterior vertebral body parts were established to be 25.75 mm (SE = 1.22) and 27.02 mm (SE = 1.06), respectively. The mean spinal canal width was set to be 22.15 mm (SE = 0.94). The mean spinal canal area was set to be 227.28 mm^2^ (SE = 18.57). All the results mentioned above and more detailed ones regarding the L2 vertebra can be found in Table 3.

**Table 3:** Statistical results of this meta-analysis regarding the L2 dimensions

L2 dimensions	Mean	Standard Error	Variance	Lower Limit	Upper Limit	Z-Value	*p*-Value
End-plate depth upper (mm)	32.12	1.09	1.19	29.98	34.26	29.42	0.00
End-plate width upper (mm)	42.98	1.57	2.45	39.92	46.05	27.45	0.00
End-plate depth lower (mm)	32.91	1.17	1.37	30.62	35.20	28.14	0.00
End-plate width lower (mm)	46.10	2.12	4.49	41.95	50.26	21.77	0.00
Vertebral body height anterior (mm)	25.75	1.22	1.48	23.36	28.13	21.14	0.00
Vertebral body height posterior (mm)	27.02	1.06	1.13	24.94	29.09	25.47	0.00
End-plate area upper (mm^2^)	1017.74	61.40	3770.31	897.40	1138.09	16.57	0.00
End-plate area lower (mm^2^)	1120.01	60.84	3701.50	1000.76	1239.25	18.41	0.00
End-plate inclination transverse plane upper (degrees)	3.66	0.24	0.06	3.18	4.13	15.12	0.00
End-plate inclination transverse plane lower (degrees)	−0.09	0.86	0.73	−1.77	1.59	−0.11	0.91
Spinal canal width (mm)	22.15	0.94	0.87	20.32	23.98	23.69	0.00
Spinal canal depth (mm)	15.08	1.00	1.00	13.13	17.04	15.12	0.00
Spinal canal area (mm^2^)	227.28	18.57	345.01	190.87	263.68	12.24	0.00
Pedicle width (mm)	8.23	0.20	0.04	7.84	8.61	41.96	0.00
Pedicle height (mm)	14.90	0.19	0.04	14.53	15.26	79.04	0.00
Pedicle width right (mm)	7.64	0.42	0.17	6.82	8.45	18.40	0.00
Pedicle height right (mm)	15.31	0.73	0.53	13.88	16.74	20.99	0.00
Pedicle width left (mm)	8.02	0.45	0.21	7.13	8.91	17.67	0.00
Pedicle height left (mm)	15.55	0.72	0.51	14.15	16.95	21.73	0.00
Pedicle area right (mm^2^)	78.05	4.82	23.22	68.61	87.50	16.20	0.00
Pedicle area left (mm^2^)	81.87	4.80	23.08	72.45	91.28	17.04	0.00
Pedicle inclination sagittal plane right (degrees)	12.21	1.64	2.69	9.00	15.43	7.45	0.00
Pedicle inclination transverse plane right (degrees)	−0.29	1.54	2.36	−3.30	2.72	−0.19	0.85
Pedicle inclination sagittal plane left (degrees)	−0.36	3.79	14.37	−7.79	7.07	−0.10	0.92
Pedicle inclination transverse plane left (degrees)	−1.07	0.96	0.92	−2.95	0.81	−1.11	0.27
Spinous process length (mm)	63.37	4.67	21.79	54.22	72.51	13.58	0.00
Transverse process width (mm)	75.07	2.88	8.32	69.41	80.72	26.03	0.00

L3 Dimensions: The mean depth of the upper part of the end-plate was found to be 33.17 mm (SE = 1.07), while its width was set at 44.82 mm (SE = 1.62). The mean depth of the lower part of the end-plate was found to be 33.76 mm (SE = 1.11), while its width was set at 49.12 mm (SE = 2.05). The heights of the anterior and posterior vertebral body parts were established to be 26.83 mm (SE = 1.16) and 26.67 mm (SE = 1.02), respectively. The mean spinal canal width was set to be 22.62 mm (SE = 0.80). The mean spinal canal area was set to be 213.87 mm^2^ (SE = 24.17). All the results mentioned above and more detailed ones regarding the L3 vertebra can be found in Table 4.

**Table 4:** Statistical results of this meta-analysis regarding the L3 dimensions

L3 dimensions	Mean	Standard Error	Variance	Lower Limit	Upper Limit	Z-Value	*p*-Value
End-plate depth upper (mm)	33.17	1.07	1.14	31.08	35.26	31.09	0.00
End-plate width upper (mm)	44.82	1.62	2.63	41.65	48.00	27.65	0.00
End-plate depth lower (mm)	33.76	1.11	1.22	31.59	35.93	30.50	0.00
End-plate width lower (mm)	49.12	2.05	4.20	45.11	53.14	23.97	0.00
Vertebral body height anterior (mm)	26.83	1.16	1.35	24.55	29.11	23.06	0.00
Vertebral body height posterior (mm)	26.67	1.02	1.04	24.67	28.67	26.13	0.00
End-plate area upper (mm^2^)	1132.32	63.14	3986.50	1008.57	1256.07	17.93	0.00
End-plate area lower (mm^2^)	935.91	401.38	161103.86	149.22	1722.59	2.33	0.02
End-plate inclination transverse plane upper (degrees)	1.93	0.13	0.02	1.67	2.19	14.55	0.00
End-plate inclination transverse plane lower (degrees)	2.11	0.18	0.03	1.76	2.46	11.94	0.00
Spinal canal width (mm)	22.62	0.80	0.64	21.05	24.19	28.25	0.00
Spinal canal depth (mm)	14.69	0.93	0.86	12.87	16.51	15.86	0.00
Spinal canal area (mm^2^)	213.87	24.17	583.98	166.51	261.24	8.85	0.00
Pedicle width (mm)	9.71	0.18	0.03	9.35	10.07	53.24	0.00
Pedicle height (mm)	14.52	0.17	0.03	14.19	14.85	85.78	0.00
Pedicle width right (mm)	9.56	0.50	0.25	8.57	10.55	18.93	0.00
Pedicle height right (mm)	15.31	0.68	0.47	13.97	16.65	22.45	0.00
Pedicle width left (mm)	9.77	0.54	0.29	8.72	10.82	18.22	0.00
Pedicle height left (mm)	14.36	0.73	0.53	12.93	15.79	19.67	0.00
Pedicle area right (mm^2^)	96.10	6.08	36.98	84.18	108.01	15.80	0.00
Pedicle area left (mm^2^)	101.67	6.72	45.12	88.50	114.83	15.14	0.00
Pedicle inclination sagittal plane right (degrees)	18.79	1.04	1.08	16.75	20.82	18.06	0.00
Pedicle inclination transverse plane right (degrees)	−0.93	1.40	1.96	−3.68	1.81	−0.67	0.50
Pedicle inclination sagittal plane left (degrees)	−2.95	8.26	68.25	−19.15	13.24	−0.36	0.72
Pedicle inclination transverse plane left (degrees)	−2.83	2.90	8.42	−8.52	2.86	−0.98	0.33
Spinous process length (mm)	65.67	4.19	17.53	57.47	73.88	15.69	0.00
Transverse process width (mm)	83.59	3.39	11.52	76.94	90.24	24.63	0.00

L4 Dimensions: The mean depth of the upper part of the end-plate was found to be 33.83 mm (SE = 1.19), while its width was set at 47.50 mm (SE = 1.87). The mean depth of the lower part of the end-plate was found to be 34.07 mm (SE = 0.95), while its width was set at 50.63 mm (SE = 1.90). The heights of the anterior and posterior vertebral body parts were established to be 26.86 mm (SE = 1.08) and 25.76 mm (SE = 1.03), respectively. The mean spinal canal width was set to be 23.19 mm (SE = 0.82). The mean spinal canal area was set to be 215.45 mm^2^ (SE = 35.98). All the results mentioned above and more detailed ones regarding the L4 vertebra can be found in Table 5.

**Table 5:** Statistical results of this meta-analysis regarding the L4 dimensions

L4 dimensions	Mean	Standard Error	Variance	Lower Limit	Upper Limit	Z-Value	*p*-Value
End-plate depth upper (mm)	33.83	1.19	1.42	31.49	36.16	28.39	0.00
End-plate width upper (mm)	47.50	1.87	3.49	43.84	51.16	25.42	0.00
End-plate depth lower (mm)	34.07	0.95	0.90	32.21	35.94	35.83	0.00
End-plate width lower (mm)	50.63	1.90	3.59	46.92	54.35	26.71	0.00
Vertebral body height anterior (mm)	26.86	1.08	1.18	24.73	28.98	24.76	0.00
Vertebral body height posterior (mm)	25.76	1.03	1.06	23.74	27.78	25.01	0.00
End-plate area upper (mm^2^)	1151.43	72.55	5263.57	1009.23	1293.63	15.87	0.00
End-plate area lower (mm^2^)	1199.99	69.64	4849.94	1063.50	1336.49	17.23	0.00
End-plate inclination transverse plane upper (degrees)	4.21	0.29	0.08	3.65	4.77	14.74	0.00
End-plate inclination transverse plane lower (degrees)	3.24	0.32	0.10	2.62	3.86	10.25	0.00
Spinal canal width (mm)	23.19	0.82	0.67	21.59	24.79	28.35	0.00
Spinal canal depth (mm)	15.06	1.04	1.09	13.01	17.10	14.41	0.00
Spinal canal area (mm^2^)	215.45	35.98	1294.25	144.94	285.96	5.99	0.00
Pedicle width (mm)	11.52	0.24	0.06	11.05	11.99	48.26	0.00
Pedicle height (mm)	14.20	0.34	0.12	13.53	14.87	41.72	0.00
Pedicle width right (mm)	11.98	1.24	1.54	9.54	14.41	9.65	0.00
Pedicle height right (mm)	15.49	0.66	0.43	14.20	16.78	23.55	0.00
Pedicle width left (mm)	12.59	1.27	1.62	10.10	15.09	9.90	0.00
Pedicle height left (mm)	15.13	0.59	0.35	13.98	16.29	25.65	0.00
Pedicle area right (mm^2^)	104.44	5.95	35.38	92.78	116.10	17.56	0.00
Pedicle area left (mm^2^)	114.18	5.98	35.76	102.46	125.90	19.10	0.00
Pedicle inclination sagittal plane right (degrees)	13.90	2.20	4.83	9.59	18.20	6.32	0.00
Pedicle inclination transverse plane right (degrees)	−2.96	3.84	14.75	−10.49	4.56	−0.77	0.44
Pedicle inclination sagittal plane left (degrees)	−3.46	5.63	31.72	−14.50	7.57	−0.62	0.54
Pedicle inclination transverse plane left (degrees)	−4.31	3.08	9.50	−10.35	1.73	−1.40	0.16
Spinous process length (mm)	63.36	4.37	19.08	54.80	71.92	14.51	0.00
Transverse process width (mm)	79.31	4.95	24.48	69.61	89.01	16.03	0.00

L5 Dimensions: The mean depth of the upper part of the end-plate was found to be 34.09 mm (SE = 0.91), while its width was set at 48.81 mm (SE = 1.91). The mean depth of the lower part of the end-plate was found to be 33.18 mm (SE = 1.18), while its width was set at 49.82 mm (SE = 2.01). The heights of the anterior and posterior vertebral body parts were established to be 27.44 mm (SE = 1.16) and 24.11 mm (SE = 0.70), respectively. The mean spinal canal width was set to be 26.46 mm (SE = 0.89). The mean spinal canal area was set to be 245.12 mm^2^ (SE = 32.12). All the results mentioned above and more detailed ones regarding the L5 vertebra can be found in Table 6.

**Table 6:** Statistical results of this meta-analysis regarding the L5 dimensions

L5 dimensions	Mean	Standard Error	Variance	Lower Limit	Upper Limit	Z-Value	*p*-Value
End-plate depth upper (mm)	34.09	0.91	0.82	32.32	35.86	37.66	0.00
End-plate width upper (mm)	48.81	1.91	3.63	45.08	52.55	25.61	0.00
End-plate depth lower (mm)	33.18	1.18	1.40	30.87	35.50	28.07	0.00
End-plate width lower (mm)	49.82	2.01	4.03	45.88	53.75	24.82	0.00
Vertebral body height anterior (mm)	27.44	1.16	1.34	25.17	29.72	23.67	0.00
Vertebral body height posterior (mm)	24.11	0.70	0.49	22.74	25.49	34.29	0.00
End-plate area upper (mm^2^)	1182.51	65.77	4325.64	1053.60	1311.41	17.98	0.00
End-plate area lower (mm^2^)	1131.35	61.99	3842.36	1009.86	1252.85	18.25	0.00
End-plate inclination transverse plane upper (degrees)	5.71	2.02	4.10	1.75	9.68	2.82	0.00
End-plate inclination transverse plane lower (degrees)	8.20	3.72	13.85	0.90	15.49	2.20	0.03
Spinal canal width (mm)	26.46	0.89	0.79	24.71	28.20	29.76	0.00
Spinal canal depth (mm)	15.69	1.37	1.88	13.00	18.37	11.44	0.00
Spinal canal area (mm^2^)	245.12	32.12	1031.70	182.17	308.08	7.63	0.00
Pedicle width (mm)	14.58	1.03	1.07	12.55	16.60	14.10	0.00
Pedicle height (mm)	14.73	1.06	1.11	12.67	16.80	13.96	0.00
Pedicle width right (mm)	15.68	1.15	1.32	13.42	17.93	13.63	0.00
Pedicle height right (mm)	18.05	0.96	0.92	16.17	19.93	18.79	0.00
Pedicle width left (mm)	15.93	1.34	1.80	13.30	18.56	11.88	0.00
Pedicle height left (mm)	18.16	1.00	1.00	16.20	20.11	18.20	0.00
Pedicle area right (mm^2^)	161.32	11.45	131.20	138.87	183.77	14.08	0.00
Pedicle area left (mm^2^)	168.23	10.07	101.35	148.50	187.96	16.71	0.00
Pedicle inclination sagittal plane right (degrees)	21.24	2.44	5.95	16.46	26.02	8.71	0.00
Pedicle inclination transverse plane right (degrees)	−2.85	2.83	7.99	−8.39	2.69	−1.01	0.31
Pedicle inclination sagittal plane left (degrees)	−8.54	6.96	48.50	−22.18	5.11	−1.23	0.22
Pedicle inclination transverse plane left (degrees)	−3.79	3.18	10.09	−10.01	2.44	−1.19	0.23
Spinous process length (mm)	59.47	4.80	23.06	50.06	68.88	12.38	0.00
Transverse process width (mm)	85.91	4.08	16.68	77.90	93.91	21.04	0.00

The results regarding anterior vertebral body height and interpedicular distance for each vertebra with respect to the patients’ gender are demonstrated in Table 7.

**Table 7:** Statistical results of this meta-analysis regarding the lumbar vertebrae dimensions with respect to patients’ gender

Male	Mean	Standard Error	Variance	Lower Limit	Upper Limit	Z-Value	*p*-Value
L1 Anterior vertebral body height (mm)	23.66	0.46	0.21	22.76	24.56	51.45	0.00
L2 Anterior vertebral body height (mm)	24.63	0.89	0.80	22.87	26.38	27.52	0.00
L3 Anterior vertebral body height (mm)	25.90	0.15	0.02	25.60	26.19	172.83	0.00
L4 Anterior vertebral body height (mm)	25.87	0.08	0.01	25.73	26.02	343.66	0.00
L5 Anterior vertebral body height (mm)	26.92	0.56	0.31	25.83	28.02	48.09	0.00
L1 Interpedicular distance (mm)	21.81	0.12	0.01	21.58	22.05	178.65	0.00
L2 Interpedicular distance (mm)	22.96	0.36	0.13	22.25	23.67	63.06	0.00
L3 Interpedicular distance (mm)	24.51	0.14	0.02	24.23	24.79	170.28	0.00
L4 Interpedicular distance (mm)	26.71	0.92	0.86	24.90	28.52	28.88	0.00
L5 Interpedicular distance (mm)	30.65	3.36	11.29	24.07	37.24	9.12	0.00

Female	Mean	Standard Error	Variance	Lower Limit	Upper Limit	Z-Value	p-Value
L1 Anterior vertebral body height (mm)	23.17	0.03	0.00	23.12	23.22	893.77	0.00
L2 Anterior vertebral body height (mm)	24.66	0.02	0.00	24.62	24.69	1367.28	0.00
L3 Anterior vertebral body height (mm)	25.53	0.61	0.38	24.32	26.73	41.52	0.00
L4 Anterior vertebral body height (mm)	25.77	0.98	0.97	23.83	27.70	26.16	0.00
L5 Anterior vertebral body height (mm)	26.67	1.44	2.09	23.84	29.51	18.46	0.00
L1 Interpedicular distance (mm)	20.95	0.27	0.08	20.41	21.49	76.48	0.00
L2 Interpedicular distance (mm)	21.85	0.47	0.22	20.93	22.77	46.52	0.00
L3 Interpedicular distance (mm)	23.39	0.08	0.01	23.24	23.55	298.45	0.00
L4 Interpedicular distance (mm)	25.80	1.11	1.24	23.61	27.98	23.14	0.00
L5 Interpedicular distance (mm)	29.27	3.18	10.14	23.03	35.52	9.19	0.00

## Discussion

Lumbar vertebrae possess distinct characteristics that set them apart from cervical or thoracic vertebrae. One notable difference is the presence of a large vertebral body. In comparison to the size of the vertebra, the spinous process is relatively short and thick, protruding perpendicularly from the body. The articular facets exhibit a distinctive vertical orientation, with the superior facets directed posteromedially and medially. Additionally, the facets possess a unique curved articular surface, which distinguishes them from the thoracic vertebrae. Another distinguishing feature is the presence of the mammillary process on the posterior aspect of the superior articular process. The height of the lumbar intervertebral discs falls between that of the cervical and thoracic intervertebral discs [21]. The present meta-analysis aimed to analyze the general morphometric properties of the lumbar spine in individuals without pathological changes. Our results represent the normal average properties of the lumbar vertebrae, which may be used as reference points when trying to diagnose potential spine pathologies.

The embryological development of the vertebrae is highly complex. Ossification of vertebrae initiates during the embryonic stage of development, approximately at 8 weeks of gestation. This process involves three primary ossification centers: one in the endochondral centrum (which forms the vertebral body) and one in each neural process (which forms the pedicles). The ossification begins at the thoracolumbar junction and progresses both cranially and caudally. The fusion between the neural processes and the centrum occurs between the ages of three and six. During puberty, five secondary ossification centers emerge. These centers appear at the tip of the spinous process, both transverse processes and on the superior and inferior surfaces of the vertebral body. The ossification centers located on the vertebral body contribute to the superior-inferior growth of the vertebrae. Ossification reaches completion around the age of 25 [19, 35].

The morphometric properties of the pedicles are especially relevant clinically. The pedicles are pieces of bone that connect the arch to the body of the vertebrae. Knowledge concerning their anatomy is of high relevance when picking the correct size of pedicle screws during pedicle screw fixation. The width of the pedicles was shown to be larger in the inferior parts of the lumbar spine when compared to the superior ones (L1 = 7.51 mm, L5 = 14.58 mm). However, numerous studies have reported variable results regarding the height of the pedicles. In the cadaveric study conducted by Arora et al. [4], where the lumbar vertebrae of 25 males and 30 females were analyzed, it was demonstrated that the pedicle height gradually increased from L1 to the L5 level. Similar results were presented by Seema et al. [29], where the lumbar vertebrae of 100 radiographs were analyzed. Contrastingly, the CT-based study conducted by Singh et al. [30] and Alam et al. [3], based on 302 and 49 patients, respectively, showed that the pedicle height decreased from L1 to L5. Interestingly, in another CT study conducted by Wolf et al. [36], it was shown that the height of the pedicles decreased from L1 to L3, then increased at L4 and L5. Our results demonstrate that the pedicle height decreased from L1 to L4, with a slight increase at L5 (14.73 mm). No significant changes between the measurements of the left and right pedicles were found. This data may be highly useful for surgeons performing pedicle screw fixation. A mismatch between the size of the screw and the pedicle may lead to severe complications, including cortex perforation, fracture of the pedicle, and even loosening of the screws [29].

The end-plate dimensions of all the lumbar vertebrae were also analyzed. The mean depth and width of both upper and lower end-plates showed a gradual increase from L1 to L5. In particular, the results of the present meta-analysis demonstrate that the mean upper and lower end plate depths in L1 were found to be 31.04 mm and 32.27 mm, respectively- while the upper and lower end-plate width were 41.09 mm and 43.97 mm, respectively. In L5 vertebrae, these mean dimensions for upper and lower end-plates were 34.09 mm and 33.18 mm, and 48.81 mm and 49.82 mm, respectively. This data is particularly relevant for procedures involving vertebral body replacement or augmentation, such as vertebroplasty and kyphoplasty. Having adequate knowledge regarding the normal dimensions of end-plates can aid in designing and selecting appropriate implants and prostheses, ensuring better fit and stability [7, 32].

The spinal canal dimensions, including width and area, were thoroughly analyzed. The results of the current study showcase a progressive increase in the spinal canal width and area from L1 to L5, which is consistent with the spinal anatomy’s natural adjustment to the spinal cords’ progression to the cauda equina. In L1 vertebrae, the mean spinal canal width was found to be 22.04 mm, while in L5 it was 26.46 mm. This data is crucial for surgical planning, especially in decompression surgeries, such as laminectomy and spinal canal decompression [8, 37]. Knowing the exact dimensions of the lumbar vertebrae may help in avoiding over-decompression, which can lead to spinal instability, and under-decompression, which might not relieve symptoms effectively [25, 37]. Finally, the transverse processes show a similar pattern, becoming considerably wider near the end of the lumbar vertebrae (L1 = 68.08 mm, L5 = 85.91 mm).

Numerous studies have analyzed the differences in the anatomy of the lumbar spine between females and males and between various ethnicities. In a study conducted by Singh et al. [30], the morphometry of the lumbar spine was analyzed in 302 Indian patients. In the study, all the measurements of the lumbar vertebrae were higher in males than in females. Contrastingly, in a study by Alam et al. [3], it was shown that the sagittal measurements of pedicle inclination (expressed in degrees) were greater in females than in males. The results of the present meta-analysis demonstrate that males had higher mean values for all of the morphometric measurements, except for the L2 anterior vertebral body height, which was slightly higher in females (24.66 mm). Tan et al. [32] conducted a study on the quantitative anatomy of the lumbar spine on 60 lumbar vertebrae from 12 Asian (Singaporean) cadavers. In the study, they compared their results with the results from a study by Panjabi et al. [25], which was carried out on Caucasian subjects. Interestingly, in contrast to the relatively larger physique of Caucasians when compared to Asians, the comparison unveiled that Asians exhibited larger quantitative dimensions for numerous morphological values.

The lumbar region of the spine is frequently subjected to surgical interventions for back and spine conditions. Common procedures in this area include surgery for herniated nucleus pulposus of the spine, lumbar discectomy, hernia excision, and vertebrae reconstruction following fractures or other reasons. A comprehensive understanding of the anatomy and dimensions of this region is critical for orthopedic surgeons and neurosurgeons to perform successful operations without complications. Precise knowledge of lumbar vertebrae dimensions, in conjunction with techniques like CT scans or radiographs, can aid in selecting the appropriate prosthesis for each patient. Percutaneous endoscopic transforaminal discectomy, particularly the Yeung endoscopic spine system (YESS) technique, involves accessing the intervertebral disc using the Kambin triangle method [11, 26]. This method encompasses reaching the exiting nerve root (hypotenuse), the upper endplate of the lower vertebra (base), and the superior articular process of the lower segment (height). During the procedure, a rongeur tool removes the nucleus pulposus by inserting it through the annulus fibrosus [24]. This technique is used for various indications, ranging from a single lumbar disc herniation to lumbar spinal stenosis, lumbar metastatic tumors, lumbar discal cysts, and revision surgeries for recurrent lumbar disc herniation [2]. Having adequate knowledge about the morphometric properties of the vertebrae may aid in adequate navigation through the spinal region during this procedure and, with that, decrease potential intraoperative complications.

The degeneration of the intervertebral disc is a significant contributor to chronic lumbar pain syndrome, which greatly affects the global working population [5, 17, 31]. To avoid fusion as a treatment approach, extensive research has focused on investigating total disc replacement. When conducting such procedures, it is crucial to be aware of the anatomical relationships to minimize the risk of complications. Recently, a second-generation lumbar prosthesis has been introduced, designed to provide control of mobility in all directions. This advancement aims to enhance the effectiveness of disc replacement procedures and improve patient outcomes [28]. Knowing the normal morphometric properties of the lumbar vertebrae, especially the vertebral bodies, may be of incredible use when performing lumbar disc replacement surgeries.

The present study is not without limitations. The accuracy of the data taken from various publications limits the results of this meta-analysis. The results acquired by different researchers or using different methods may be a potential source of bias. The authors were unable to perform some of the analyses due to an insufficient amount of consistent data. Despite the extraction of data regarding patients’ mean age, weight, and height, the correlation of those data with the studied parameters was not performed to prevent the potential bias of the results. Data regarding the transitional anomalies of the lumbar spine were not considered in this study, which potentially lowers the clinical application of the present meta-analysis. Furthermore, most of the evaluated studies come from Asia, and most of the patients studied were from Asia (*n* = 1076). Therefore, the results of the present study may be burdened with a potential bias, as they may reflect the anatomical features of Asian people rather than the global population. Although not without limitations, our meta-analysis attempts to estimate lumbar vertebrae anatomy based on data from the literature that meets the requirements of evidence-based anatomy.

## Conclusions

The present meta-analysis provides the most up-to-date and evidence-based normative morphometric properties of the lumbar vertebrae. The findings from this study reveal that the width of the spinal canal progressively increases towards the lower end of the lumbar spine (L1 = 22.04 mm, L5 = 26.46 mm). Additionally, the transverse processes exhibit a similar trend, significantly widening as they approach the lower lumbar vertebrae (L1 = 68.08 mm, L5 = 85.91 mm). Moreover, the pedicle height decreased from L1 to L4, with a slight increase observed at L5 (14.73 mm). No significant differences were observed between the measurements of the left and right pedicles. Our results may be of immense use for surgeons performing various spinal surgeries, such as pedicle screw fixation, percutaneous endoscopic transforaminal discectomy, or lumbar disc replacement.

## Data Availability

No datasets were generated or analysed during the current study.
